# Prototype of a novel cold snare polypectomy device

**DOI:** 10.1055/a-2651-9505

**Published:** 2025-08-08

**Authors:** Lode Van Overbeke, Gerd Jacomen, Koen Chiers

**Affiliations:** 1 218714 Department of Gastroenterology, AZ St Maarten, Mechelen, Belgium; 2 218714 Department of Pathology, AZ St Maarten, Mechelen, Belgium; 3 86797 Veterinary Pathology, UGent, Veterinary Medicine, Ghent, Belgium

There is a growing trend toward removing large sessile colonic polyps using cold snare piecemeal resection. This technique has proven to be both safer and more cost-effective than the traditional endoscopic mucosal resection method. However, cold snares are restricted to removing only small and superficial tissue fragments, increasing the risk of residual polyp tissue 1 .

We hypothesize that the safety of cold snare resection is not attributed to the superficial nature of the excision but rather to the absence of coagulation-related damage to the endothelium, vascular muscle layer, and muscularis propria.

Our objective is to develop a device capable of achieving larger and deeper resection specimens without coagulation.

To achieve this, we designed a robust 10-Fr catheter with a specially engineered cutting tip through which a conventional snare can be advanced. After the polyp is encircled by the conventional snare, the catheter is advanced, providing additional cutting capacity ( Fig. 1 ).

**Fig. 1:**
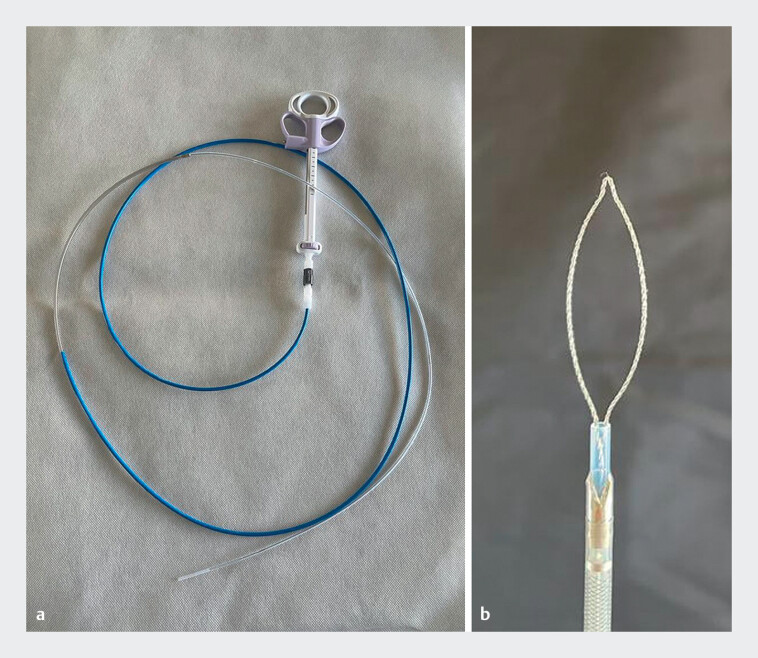
The device comprises a robust 10-Fr catheter with a specially engineered cutting tip through which a conventional snare can be advanced. After encircling the polyp by the snare, the catheter is advanced forward, providing additional cutting capacity.

We tested this prototype on an anesthetized pig where normal colonic mucosa was resected for evaluation ( Video 1 ).

Prototype of a novel cold snare polypectomy device.Video 1

A 15-mm snare device was used without submucosal injection, yielding four resections with a median diameter of 17.65 mm (range 12.8–23.4) and a depth from the muscularis mucosae of 175 µm (range 0–500). Subsequently, a 25-mm snare device was used with submucosal injection (to capture more mucosa), yielding three resections with a median diameter of 20.3 mm (range 16.0–22.8) and a depth of 400 µm (range 200–1100). One resection was performed using the 15-mm snare (without device), yielding a resection diameter of 5.6 mm and a depth of 0 µm ( Table 1 and Fig. 2 ).

**Table:** **Table 1** Results of four resections using the 15-mm snare device, three resections using the 25-mm snare device, and one resection using a 15-mm cold snare (without device).

	15-mm device	25-mm device	15-mm snare
Resection specimen diameter (mm)	12.8, 17.8, 23.4, 17.5	20.3, 22.8, 16.0	5.6
Median (range)	17.65 (12.8–23.4)	20.3 (16.0–22.8)	
Resection depth (µm)	500, 50, 300, 0	200, 1100, 400	0
Median (range)	175 (0–500)	400 (200–1100)	
Adverse events	None	None	None

**Fig. 2:**
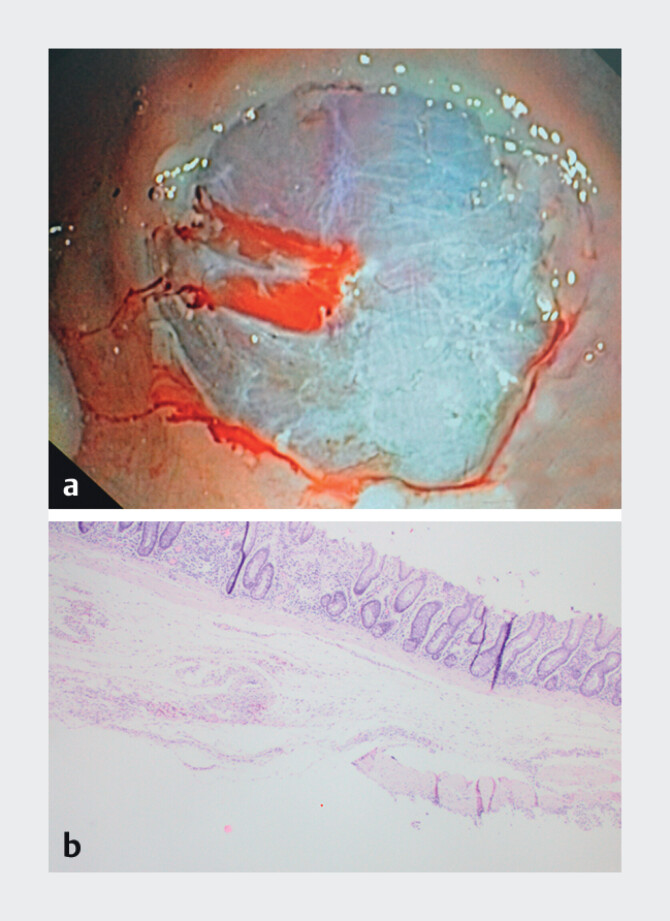
**a** Resection specimen (diameter 22.8 mm) obtained with the 25-mm device. **b** Histopathological examination reveals a large fragment containing mucosa, muscularis mucosae, and substantial submucosa including an arteriole (1100 µm).

No perforations or significant acute bleeding events were observed. Since the pig was euthanized following the procedure, no conclusions could be drawn regarding delayed bleeding.

Endoscopy_UCTN_Code_TTT_1AQ_2AD_3AB

## Notes

Endoscopy E-Videos https://eref.thieme.de/e-videos
*E-Videos* is an open access online section of the journal *Endoscopy* , reporting on interesting cases and new techniques in gastroenterological endoscopy. All papers include a high-quality video and are published with a Creative Commons CC-BY license. Endoscopy E-Videos qualify for HINARI discounts and waivers and eligibility is automatically checked during the submission process. We grant 100% waivers to articles whose corresponding authors are based in Group A countries and 50% waivers to those who are based in Group B countries as classified by Research4Life (see: https://www.research4life.org/access/eligibility/ ). This section has its own submission website at https://mc.manuscriptcentral.com/e-videos .
